# The Effect of Freezing Sheep’s Milk on the Meltability, Texture, Melting and Fat Crystallization Profiles of Fresh Pasta Filata Cheese

**DOI:** 10.3390/ani11092740

**Published:** 2021-09-19

**Authors:** Jakub Biegalski, Dorota Cais-Sokolińska, Jolanta Tomaszewska-Gras, Hanna M. Baranowska

**Affiliations:** 1 Department of Dairy Products Quality, Faculty of Food Science and Nutrition, Poznań University of Life Sciences, ul. Wojska Polskiego 31, 60-624 Poznań, Poland; jakub.biegalski@up.poznan.pl; 2 Department of Food Safety and Quality Management, Poznań University of Life Sciences, ul. Wojska Polskiego 31, 60-624 Poznań, Poland; jolanta.tomaszewska-gras@up.poznan.pl; 3 Department of Physics and Biophysics, Poznań University of Life Sciences, ul. Wojska Polskiego 38/42, 60-637 Poznań, Poland; hanna.baranowska@up.poznan.pl

**Keywords:** frozen milk, sheep’s milk, pasta filata cheese, meltability, water mobility

## Abstract

**Simple Summary:**

Sheep’s milk is usually produced on small farms. It is mainly used in the pro duction of cheese products. One of the methods of extending the shelf life of sheep’s milk is freezing it. In this study we examined the effect of freezing on sheep’s milk and a mixture of sheep’s and cow’s milk on the quality of fresh pasta filata cheeses produced from the milk. It has been proven that the freezing of milk affects the possibility of using it in later cheese processing. Freezing sheep’s milk influenced, among others, a greater hardness and less elasticity of the cheese. We also noticed that the addition of frozen sheep’s milk caused consumer dissatisfaction.

**Abstract:**

Sheep’s milk is produced in smallholdings, which hinders the continuity of production. Therefore, freezing during periods of high production can be a solution. Herein, we examined the effect of freezing on sheep’s milk and a mixture of sheep and cow’s milk (70:30, *v/v*) on the quality of fresh pasta filata cheeses produced from the milk. Frozen/thawed sheep’s milk contributes little to the development of innovative and reformulated cheeses. This was due to 24% higher hardness and greater extensibility and cutting force, as well as lower stretching and elasticity. Although their flowability increased (Oiling-off from 3 to 12%), the meltability (tube test, and Schreiber test) decreased. Additionally, the use of frozen milk caused consumer dissatisfaction. The consumer penalty analysis of the just–about–right showed that freezing of the milk caused the loss of the refreshing, elasticity and shininess of pasta filata cheeses.

## 1. Introduction

The supply of sheep’s milk in many regions depends on low sheep productivity, the seasonality of milk production and the short period of lactation. In many countries, sheep’s milk is produced in smallholdings, which hinders large-scale production [1]. The largest amounts of sheep’s milk are produced in Asia (46.3%) followed by Europe (29.8%) and Africa (23.0%). Turkey produces the greatest amount of fresh sheep’s milk, followed by China (mainland) and Greece. Approximately 10.6 Mt of whole, fresh sheep’s milk is produced globally [2]. Moreover, sheep’s milk production is expected to increase by 26% (+2.7 Mt) by 2030 [3] due to the growing demand for cheese and its use in infant formulas and nutraceuticals [4,5]. For many consumers, sheep’s milk and its products are considered essential for the proper functioning of the human body, and are perceived by consumers as a material with high health-promoting potential [6,7]. Therefore, in our opinion the dairy sector may also be interested in mixing sheep’s milk with cow’s milk. Due to the development of appropriate technologies for the processing of mixed milk of various mammals, it is possible to maintain or increase the amount of bioactive ingredients that are contained within [8,9]. Moreover, the use of milk mixtures from different species of mammals is consider an interesting approach to propagate dairy processing in many regions of the world [10].

Sheep’s milk contains an average of 5.7% protein, 7.4% fat, 4.8% lactose and 0.9% ash [11]. The protein fractions of sheep’s milk are mainly: casein (41–46 g/kg) and whey proteins (8–16 g/kg), which is in contrast to the protein fractions of cow’s milk (24–28 g/kg caseins, 5–7 g/kg whey proteins) [11,12]. Sheep’s milk casein micelles have a higher degree of mineralization due to greater amount of calcium, reduced hydration and thermal stability compared to cow’s milk micelles [13]. Overall, the calcium content of sheep’s milk is higher (1.59–2.42 g/L) than that of cow’s milk (0.90–1.84 g/L). The share of short and medium-length saturated fatty acids (FA) such as caproic (C6:0), caprylic (C8:0), capric (C10:0) and lauric (C12:0) in sheep’s milk fat is much higher (14.2–17.7%) than in cow’s milk (9.0–11.0%) [4,12]. Furthermore, sheep’s milk fat has low ratio of omega-6 to omega-3 (*n*-6: *n*-3) FA, which is associated with the prevention of cardiovascular diseases and cancer. Sheep’s milk may contain higher amounts of omega(n)-3, polyunsaturated fatty acid (PUFA) and biohydrogenation intermediate PUFA, including conjugated linoleic acid and trans-mono-unsaturated FA, vaccenic acid, rumenic acid and branched FA compared to cow’s milk [12]. The high CLA content in sheep’s milk, compared to the milk of other ruminants, is of particular interest for the prevention of cancer diseases. It is also known that sheep’s milk contains higher amounts of vitamin A (40–84 µg/100 g) and D (0.18 µg/100 g) than cow’s milk (29–52 µg/100 g and 0.03–1 µg/100 g, respectively) [12]. The characteristic composition of sheep’s milk and its role in many nutritional interventions make it increasingly sought after by consumers.

However, the limited supply of sheep’s milk necessitates the collection of this raw material and the use of alternative storage techniques to ensure the continuity of production in the dairy plant. A general solution is to freeze sheep’s milk. The freezing time strictly depends on the cooling rate, the initial quality of the milk used in production and the final storage temperature [14]. Milk should be immediately chilled and frozen as soon as possible in order to reduce the growth of microorganisms and enzyme activity [15]. The freezing process can significantly affect the fat globules, promoting the release of lipoproteins and diminish stability of the lipid phase [14,15]. Additionally, the process increases the particle size, which leads to the coalescence and natural separation of the cream, as well as an increased rate of fat oxidation and the occurrence of lipolysis [16,17]. Freezing can also greatly affect the destabilization of proteins with a micellar nature, which depend on the temperature. For example, the destabilization of sheep’s milk proteins at −15 °C occurs after 6 months of storage, and at −27 °C after 12 months [18]. The destabilization of milk proteins consequently leads to casein aggregation and reduction of the water holding capacity (WHC). This is directly influenced by the breaking of hydrogen bonds between polypeptides [1].

However, to date, there are no studies that examine the influence of freezing on sheep’s milk or a mixture with cow’s milk, and its effects on the quality of fresh pasta filata cheeses prepared from the milk. Albenzio et al. [19] and Tripaldi et al. [20] described the growing interest in sheep’s milk pasta filata cheese, even though sheep’s milk generally has poor stretching ability [21]. Herein, we examined whether freezing sheep’s milk can cause changes in the texture, meltability, water activity, water mobility, crystallization, melting and thermal oxidation of fat. Differential scanning calorimetry (DSC) was used to elaborate the water−protein−fat interactions in pasta filata cheeses, and nuclear magnetic resonance (NMR) relaxation techniques were used to assess the mobility of water. We present these properties on the background of sensory attractiveness from the consumers’ perception, which was investigated in detail.

## 2. Materials and Methods

### 2.1. Sheep’s and Cow’s Milk Samples

The research material was fresh, raw, full sheep’s milk collected from Polish Dairy sheep line 05 (average body weight of ewes was 60 kg ± 5.5 kg and aged 3, 4 and 5 years) purchased from local farmers in Greater Poland (52 L). Milk was collected from sheep in the months from May to September 2020. Half of the milk was frozen in 5 L PP bottles (milk volume up to 4.5 L, Ø = 16 cm, h = 40 cm) at −24 °C in Fiocchetti Super-Polo freezer (C.F. di Ciro Fiocchetti & C. s.n.c., Luzzara, Italy). Storage time was 12 weeks. The milk was thawed immediately before further processing. Milk from Holstein-Friesian cows was also used. It was high hygienic and cytological quality milk intended for dairy plants. A total of 260 L of sheep’s milk and 260 L of cow’s milk were used.

### 2.2. Mixture of Milk

Cow’s milk was combined in the proportion of 70:30 (*v*/*v*) with fresh sheep’s milk and in the same proportion with frozen milk after thawing.

### 2.3. Cheese Preparation Protocols

The production of pasta filata cheese involves normalization and pasteurization of milk or a mixture of cow’s and sheep’s milk, and production of the curd and its acidification, followed texturization of the acidified curd, which involves heating, kneading, and stretching by soaking the curd in hot water. The milk or milk mixture was pasteurized (75 °C, 15 s) in MILKY FJ15 (Franz Janschitz GmbH, Althofen, Austria). After cooling to 40 °C, thermophilic starter culture Lyofast SAB 440B of Sacco (Cadorago, Italy) was added to the milk in an amount of 8 UC (dosage units) to 20 L of milk. The starter culture consisted of: *Streptococcus thermophilus*, *Lactobacillus acidophilus* and *Bifidobacterium animalis* subsp. *lactis*. At pH 6.5, Beaugel 5 rennet (Ets COQUARD, Villefranche Sur Saône, France) with a chymosin activity above 150 mg/L at a strength of 1/3000 was added in the amount of 14 mL per 20 L of milk. This amount was used to form the curd after 30 min. The curd was cut into 1 × 1 cm cubes and set aside for 20 min until pH 5.9 was reached. The cheese curd was drained at 23 °C for 2 h to pH 5.2. Plasticization (kneading and stretching) was carried out in water with salt (77–80 °C). The formed cheese was cooled in water with salt (16%, 12 °C). The cheese was packed with a brine and stored at 3 °C in the form of spheres with a weight of 220 g. The range of changes of cheese quality features of the model were rated after 2 days of manufacturing at 3 ± 0.5 °C storage.

Test specimens were taken from different production batches (*n* = 5). Approximately 3.8 kg cheese (17 balls) was obtained from 20 L of milk. Each ball of cheese was 220 g. The cheese was prepared in pilot plant scale using pilot industrial equipment, and each batch was analyzed twice.

### 2.4. Composition and Freezing Point

A Bentley DairySpec FT Manual (Bentley Instruments, Inc., Chaska, MN, USA) was used to determine the composition of the studied milk. The composition of the cheese was determined according to moisture [22], protein, and fat [23] content. Total protein (TN−NPN) × 6.38; C = casein (TN−NCN−NPN) × 6.38; WP = whey protein (NCN−NPN) × 6.38. The freezing point of the studied milk was determined according to ISO 5764 [24] standard method using an Advanced Model 4D3 cryoscope with 3LH700 thermistor probe (Advanced Instruments Inc., Norwood, MA, USA).

### 2.5. Acidity and Conductivity

pH was measured using a CP-402 pH-meter (Elmetron, Zabrze, Poland) equipped with a IONODE IJ44A electrode (Ionode Pty. Ltd., Tennyson, Australia). The titratable acidity values were expressed as Soxhlet-Henkel degree (°SH, 1 °SH = 0.0225 lactic acid %). Conductivity was measured using a CP-505 conductometer (Elmetron, Zabrze, Poland) equipped with a EC-60 conductometric sensor (Elmetron, Zabrze, Poland).

### 2.6. Water Activity

The water activity was measured with an AquaLab Series 4TE instrument (Decagon Devices Inc., Pullman, WA, USA). The following salt solutions were used for reference: 0.5 M KCl about a_w_ = 0.984 (15 °C), 6 M NaCl of a_w_ = 0.760 (20 °C), 8.57 M LiCl of a_w_ = 0.500 (25 °C) and 13.41 M LiCl of a_w_ = 0.250 (25 °C). Samples of v = 15 mL were placed in a DE 501 measurement vessel (Decagon Devices Inc., Pullman, WA, USA) and tested at 15 °C.

### 2.7. Viscosity

Absolute values of dynamic viscosity of the studied milk were recorded using a Hőppler KF10 viscosimeter by RheoTec Messtechnik GmbH (Ottendorf, Germany). The time (s) was measured (t) for a ball to fall over a distance of 100 mm at an inclination angle of 70° within a volume v = 40 mL. The angle constant for the measurement was FH = 0.952 (–). Balls used in the tests were made from Fe–Ni alloy with diameters of Øk3 = 15.552 mm and Øk4 = 15.199 mm and mass of mk3 = 16.0627 g and mk4 = 14.1797 g, density of dk3 = 8.156 g/mL and dk4 = 7.713 g/mL at the apparatus constants ascribed based on the certificate K3 = 0.13543 mPa∙mL/g and K4 = 1.2268 mPa∙mL/g. On the basis of sample density (dp), established using an areometer by Areometr (Warszawa, Poland) at fiducial temperature (T = 20 °C) within the range from 1015 to 1045 g/mL dynamic viscosity was calculated using Equation (1):

(1) η=t×(dk−dp)×K×F (mPa·s)

### 2.8. Texture Profile Analyses

The measurement of selected cheese texture parameters was performed using a texturometer (Stable Micro Systems Ltd., Surrey, UK) using: P/1S—firmness and stickiness (pre-test speed 1.5 mm/s, test speed 2.0 mm/s, post-test speed 10.0 mm/s, distance 5 mm), P/5—softness (pre-test speed 2.0 mm/s, test speed 1.0 mm/s, post-test speed 1.0 mm/s, distance 10 mm), A/BC—firmness (pre-test speed 0.5 mm/s, test speed 0.5 mm/s, post-test speed 10.0 mm/s, distance 25.0 mm) and A/WEG—hardness and brittleness (pre-test speed 1.0 mm/s, test speed 2.0 mm/s, post-test speed 10.0 mm/s, distance 10.0 mm). Results were recorded using Texture Exponent E32 version 4.0.9.0 software (Godalming, Surrey, UK).

### 2.9. Meltability

Meltability of cheese was determined by two methods: the Schreiber test and test tube method [25]. For the Schreiber test a circular cookie cutter, 39.5 mm in diameter, was used to cut 5 mm high disc of cheese. This disc was placed in a covered 15 mm × 100 mm thin-walled Pyrex Petri dish and heated in a forced draft oven preheated to 232 °C for 5 min. Specimen expansion was measured using a scale having six lines marked on a concentric set of circles. The Schreiber test meltability was given as the mean of six readings on the arbitrary scale of 0–10 units.

In the case of the test tube method, 10 g of grated cheese was placed in a tube (32 mm × 250 mm) and packed to form a plug at the bottom. The height of cheese was marked. The test tube was kept vertically in a refrigerator at 4 °C for 30 min and then horizontally in an oven heated at 104 °C for 60 min. Meltability was measured as flow distance in mm of melted cheese.

### 2.10. Oiling-Off

Oiling-off (fat-ring test) was determined according to the method described by Schenkel et al. [26] and Hartmann et al. [27]. Grated cheese samples were prepared and weighed (5 g) into stainless steel rings Ø = 36 mm, which were placed in a glass Petri dish on to filter paper (pore size from 5 to 13 µm, 88 s medium speed filtration according to DIN 53137, LLG-Labware, Meckenheim, Germany). After heat treatment at 100 °C for 7 min in an oven WTB Binder (Tuttlingen, Germany), the steel ring was removed. The sample was cooled for 5 min at ambient temperature and then a picture was taken. The free oil formation was expressed as the percentage of the area soaked by free oil relative to the area of the total filter paper.

### 2.11. Differential Scanning Calorimetry (DSC)

DSC was conducted using a Perkin Elmer DSC 7 differential scanning calorimeter (Perkin Elmer, Norwalk, CT, USA) equipped with an Intracooler II and running under Pyris software. Samples of cheese (9–10 mg) were weighed into aluminum pans of 20 μL (Perkin Elmer, No. 0219–0062) and hermetically sealed. The sample pan was placed in the calorimeter at 5 °C and then subjected to the following process-temperature program: (1) heating and isotherm for 5 min at 70 °C to melt all crystals and nuclei; (2) cooling at 5 °C/min to −40 °C; and (3) heating at 5 °C/min to 70 °C. The following parameters were analyzed from the first melting curve: T1_on_—onset temperature, T1, T2—peak temperatures, T1_end_—final melting temperature, and enthalpy of milk fat melting ∆H_m1_ (J/g) determined as the area limited by the melting curve and the base line. The onset temperature (T_on_) was taken at the intersection of the baseline with the tangent to the left side of the melting peak. From the second melting scan the temperatures of ice melting Ti_onset_, Ti_peak_ and enthalpy ∆H_ice_ of ice melting, calculated per 1 g of water were measured. The percentage of unfrozen water in the water fraction (UFW) was calculated using Equation (2):

(2) $$UFW=100-\left(\frac{\Delta H_{ice}}{\Delta H_{ref}}\right)\times 100\;\left(\%\right)$$

where ΔH_ice_ is the enthalpy of ice melting per unit mass of water contained in cheese (J/g), ΔH_ref_ is the enthalpy of ice melting for samples of pure water, equal to 333.7 J/g.

### 2.12. Nuclear Magnetic Resonance (NMR)

The samples of 1.5 cm diameter and 1.2 cm height were placed in measuring test tubes and sealed using Parafilm. Measurements of the spin−lattice (T_1_) and spin−spin (T_2_) relaxation times were performed using a pulse NMR spectrometer operating at 30 MHz (WL Electronics, Poland). The inversion−recovery (π–TI–π/2) impulse sequence was applied for measurements of T_1_ relaxation times. Distances between impulses (TI) were changed within the range of 4 to 800 ms and repetition time of 15 s. Each time, 32 free induction decay (FID) signals and 119 points from each FID signal were collected. Calculations of the spin−lattice relaxation time values were performed with CracSpin program using spin grouping approach [28]. Time changes of the current value of FID signal amplitude in the employed frequency of impulses were described using Equation (3):

(3) $$M_{z}\left(TI\right)=M_{0}\times \left(1-2e^{\frac{-TI}{T_{1}}}\right)$$

where M_z_(TI) is the actual magnetization value, M_0_ is the equilibrium magnetization value, TI is the distance between impulses, and T_1_ is the time of relaxation.

A monoexponential magnetization recovery was found, which means that the system relaxes with one T_1_ spin−lattice relaxation time. Measurements of T_2_ spin−spin relaxation times were taken using the pulse train of the Carr−Purcell−Meiboom−Gill spin echoes (π/2–TE/2– (π)_v_). The distance between π (TE) impulses amounted to 1 ms. The repetition time was 15 s. The number of spin echoes (n) amounted to 50. Ten accumulation signals were employed. To calculate the spin−spin relaxation time values, Equation (4) was used [29]:

(4) $$M_{x,y}\left(TE\right)=M_{0}\overset{n}{\underset{i=1}{\sum }}p_{1}e^{\frac{-TE}{T_{2i}}}\;$$

where M_x,y_(TE) is the echo amplitude; M_o_ is the equilibrium amplitude; TE is the distance between π; impulses; p_i_ is the fraction of protons relaxing with the T_2i_ spin−spin time.

### 2.13. Acceptability of Appearance and Consumer Penalty Analysis

In the sensory test, consumers (*n* = 97; ages 20 to 62; M_age_ = 34, SD = 9.57) were asked to indicate how much they liked or disliked each product on a 9-point hedonic scale (9 = like extremely; 1 = dislike extremely). Samples of 10 g (10–12 °C) were served. They were evaluated using a 5-point just-about-right (JAR) scale anchored at both extremes (1 = not enough to 5 = too much), with a central point at 3 (3 = ideal): aroma (acidity), flavor (refreshing, sweet milk), texture (elasticity, smoothness) and appearance (shininess).

### 2.14. Statistical Analyses

The influence of the composition on the samples was evaluated by one-way analysis of variance (ANOVA) followed by Tukey’s HSD post hoc test for multiple comparisons. Data were analyzed using TIBCO Statistica data analysis software, version 13.3.0 (TIBCO Software Inc., Palo Alto, CA, USA). A critical level of significance of α = 0.05 was used throughout this study.

## 3. Results and Discussion

### 3.1. Composition and Physicochemical Properties of Milk and Fresh Pasta Filata Cheese

The sheep’s milk, compared to the cow’s milk, contained more solid nonfat (by 45%, *p <* 0.05, Table 1). The casein content in the sheep’s milk was 41.8 g/kg, which was 54% more than in cow’s milk. Caboni et al. [30] showed that the casein content in sheep’s milk ranges from 36.8 to 40.8 g/kg. Additionally, they indicated that the fat/protein ratio was 0.87–1.04. Compared to our results (1.68) this was considerably lower. The different technological parameters of concern between our examined sheep’s and cow’s milk included freezing point, viscosity, density, and water activity (*p <* 0.05). Compared to our results, the Bučević-Popović et al. [31] tested sheep’s milk showed a lower density (1.0263 kg/m^3^), similar acidity (0.24% lactic acid) and lower pH 6.45. Sheep’s milk examined by Faccia et al. [32] had a pH of 6.62. In our study, the conductivity of sheep’s milk (3.85 mS/cm) was lower by almost 7% than that of cow’s milk (4.12 mS/cm). This was probably due to approximately double the fat content in sheep’s milk (86.1 g/kg) than in cow’s milk (*p <* 0.05). According to various reports, the fat content of sheep’s milk varies widely from 3.8% [33] to 6.48% [34]. Yanthi et al. [35] demonstrated that the conductivity of cow’s milk at 4.59 mS/cm possessed 3.05% fat content and 2.81% protein content. Additionally, they indicated that the ingredients had a significant effect on the conductivity of cow’s milk such as total solid, solid nonfat, lactose and freeze point deviation. Moreover, Kaşikçi et al. [36] reported that higher conductivity was related to the mineral content in milk. Factors such as milk temperature, pH and fat content also influenced the conductivity value [37]. Furthermore, differences in composition, fat, protein, vitamins and minerals affected the conductivity value. In general, conductivity is influenced, among others, by lactose [35]. Fahmid et al. [38], reported that a low lactose concentration in milk can increase the conductivity value, which is reflected in the research presented in this paper. High conductivity in milk impedes the analysis of low frequency relaxations and contributes to a significant electrode polarization in dielectric measurement, as described by Agranovich et al. [39]. We used a nuclear magnetic resonance (NMR) technique to assess the structural and dynamic importance of water.

Milk is a complex colloidal liquid exhibiting a multiscaled structure, and the differences in properties and composition between sheep’s and cow’s milk, mainly fat/protein content and proportions, are responsible for different cheese parameters. In addition, factors such as larger casein micelles, greater calcium per casein weight, and mineral content in sheep’s milk will ultimately influence the coagulation time, coagulation rate, and the amount of rennet needed [21].

The initial fluctuations in the composition of sheep’s and cow’s milk influenced the moisture content of the final cheese product (Table 2). Pasta filata cheeses from unfrozen and frozen sheep’s milk had a moisture content of 488.8 g/kg and 491.1 g/kg, respectively (*p >* 0.05). The mixture of cow’s and sheep’s milk (70:30) resulted in cheeses with a moisture content of approximately 570 g/kg. Therefore, the proportion of fat:dry matter, assuming that standardized milk was used in the experiment, ranges from 0.37 (sheep’s milk cheese) to 0.48 (cow’s milk cheese). The proportion of protein:dry matter was 0.53 and 0.44, respectively.

Our study showed the possibility of producing quality pasta filata cheeses from frozen/thawed sheep’s milk. A model curd was produced from this milk, which was acidified and kneaded in hot water. The pasta filata cheeses were visually rindless, smooth, elastic with a long-stranded, parallel-orientated fibrous protein structure without evidence of curd granules. The plastic consistency characteristic of such cheeses had been obtained [21]. Our cheeses were ascribed to the soft cheese category of Codex Alimentarius [40].

Utilization of animal milk in this manner concurs with low supply, overcoming the seasonality of milk production, low production and short lactation periods. Yu et al. [41] showed that when using goat’s milk the optimal approach to maintain the natural quality of milk involves ultra-cryogenic freezing−homeothermic thawing. According to Nurliyani et al. [42], goat’s milk should be stored at −20 °C for up to 30 days. However, in the case of sheep’s milk, storage time could be extended significantly longer. Yogurt produced from frozen sheep’s milk stored for 6 months at −20 °C did not differ in pH, acidity, lactose, appearance and color, body and texture, flavor, overall acceptability, consistency, apparent viscosity or syneresis from sheep’s unfrozen milk yoghurt [43]. Voutsinas et al. [44] brined soft cheese from ultrafiltered and frozen sheep’s milk (at −20 °C up to 6 months), which displayed a sandy texture, received lower scores for appearance, were harder and more acidic in flavor, and ranked lower in overall quality. Therefore, the production of brined soft cheese from ultrafiltered and frozen sheep’s milk does not seem viable for commercial use.

Pasta filata cheeses from sheep’s milk (samples S) had a higher (1.4) protein:fat ratio than (0.9) pasta filata fior di latte cheeses also from sheep’s milk [32]. The pH of both cheeses was similar (5.12 and 5.16, respectively). In contrast, the protein:fat ratio was more similar to that of 1.2 in pasta filata-mozzarella cheeses with moisture 581.2 and 612.7 g/kg [20]. The activities most significant in the cheese production technology, such as stretching and molding phases, have a significant effect on moisture and fat losses in the fresh curd [45].

The water activity of the cheeses was not affected by milk, but only by freezing (Table 2). Frozen sheep’s milk cheeses and cheeses from a mixture of cow’s milk with frozen sheep’s milk had higher (*p <* 0.05) water activity than other cheeses. Hence, the structural and dynamic importance of water in frozen milk was crucial. Agranovich et al. [39] examined the individual contribution of lactose, fat and protein (mainly casein) content toward the total relaxation pattern during the dielectric spectroscopy study of water dynamics in frozen bovine milk. Our study used 5 L packages for freezing, therefore, the phase transition time was as short as possible and ensured a low amount of ice crystals. Large ice crystals could damage fat globules [16] and, thus, fat loss during curd stretching in pasta filata cheese production. The longer the phase change time, the slower the formation of ice crystals, which increased migration of calcium out of the micelles, as well as the concentration of soluble calcium [46]. Thermal histories of the sheep’s milk freezing process in different packages, including 5 L packages, have been presented by Tribst et al. [47].

### 3.2. Texture Profile, Meltability and Stretching

The addition of frozen sheep’s milk to cow’s milk effected all the measured parameters of the cheeses texture (Table 3, *p <* 0.05). Extensibility force, hardness and cutting force increased. Distance at break, meaning the brittleness of pasta filata cheeses made from the mixture, decreased by 4%, while stretching decreased from 131.4 to 129.8 mm. In the case of cheeses made only from sheep’s milk, freezing of the milk did not affect stretching (*p* > 0.05). In the analysis of the texture profile of these cheeses, it was found that the freezing of the milk increased extensibility and cutting force (60% and 130%, respectively, *p <* 0.05).

The addition of sheep’s milk to cow’s milk plays an important role in creating the cheese’s texture. Reports have shown that it increased the hardness of reduced fat Muenster-type cheese [48]. Additionally, Everett and Auty [49] showed that the texture of cheeses was influenced by casein−casein, casein−water, and casein−fat interactions, the state of the water (bulk or bound to the casein matrix). Therefore, the texture parameters are important regarding the protein:fat ratio, as well as free oil formation. Addis et al. [50] found sheep’s milk cheese with increased protein content in relation to fat was more compact, cohesive and hard. To supplement the knowledge about the interactions between the components, it is worth measuring water activity and water mobility. An understanding of mobile bulk water trapped within the casein matrix and a less mobile water phase bound directly to the casein will be given. This is important for explaining the diffusion of water molecules and fat globules in cheese. In the production of pasta filata cheeses, during the texturization process (heating, kneading and stretching), the elongation of protein fibers and aggregation of fat globules and pools of fat in the direction of stretching occurs. Water channels form between the casein fibers, which also elongate [51]. Fat globules in the channels compress during protein swelling. Sheep’s milk has a lower fat globule size than cow’s milk [33]. Overall, sheep’s milk has good coagulation and cheese-making properties. Vacca et al. [52] determined the ingredient most associated with gelling, the hardening time of the curd and water retained in the curd, was lactose. Lactose strongly interacts with water, forming a water clustered structure with ~123 affected water molecules per lactose molecule [39]. Water and lactose form analogous complexes such as the interfacial ice-like water. Lactose governs the glass transition of most concentrated liquid milk products. Agranovich et al. [39] showed that with increasing competition for water, the nature of hydration around the lactose molecule changes, resulting in the water becoming even more ordered around the lactose molecule.

### 3.3. Melt/Flow Profiles of Cheeses

The freezing of milk reduces the meltability of pasta filata cheeses made from sheep’s milk or mixed sheep’s milk (Table 4, *p <* 0.05). Cheeses with the addition of nonfrozen sheep’s milk had a higher meltability than those produced from cow’s milk. When the frozen milk was added, the meltability of the cheeses in the tube test decreased (*p <* 0.05). Using only frozen sheep’s milk cheeses, reduced meltability (tube test) by 11% (*p <* 0.05) compared to that of cow’s milk cheeses. The Schreiber test did not confirm such changes. Frozen sheep’s milk cheese had 42% lower meltability (tube test) than nonfrozen sheep’s milk cheese. Addition of some frozen sheep’s milk, reduced the meltability (tube test) of the cheese by 20%. This tendency was confirmed by the results of the Schreiber test. The flowability of pasta filata cheeses made entirely of frozen sheep’s milk increased 12-fold, whereas the mixture of milks increased 4-fold. No difference was found between the flowability of the cheese from the mixture with nonfrozen milk and cow’s milk cheese (*p >* 0.05).

The meltability and flowability of cheeses are important when cheeses are ingredients in food services. Upon heating of the cheese, the visual change is its softening and flow, where the proteins flow along with the melted fat [53]. Especially in pasta filata cheeses, the texture profile and melt/flow are more important than the taste as typical functional characteristics. If the freezing process is implemented, the range of changes is quite wide. Kuo and Gunasekaran [54] examined the effect of freezing and frozen storage of pasta filata cheese. They showed that after the freezing process (with tempering), the porosity of the cheese increased. As a result of tempering, water clusters are unable to fully rebind into proteins. This is what can lead to an increase in the porosity of the protein matrix. This also resulted in an increased meltability and decreased stretchability of pasta filata Mozzarella cheese.

### 3.4. Characteristics of Water Distribution and Mobility in Cheese Based on Nuclear Magnetic Resonance (NMR) and Differential Scanning Calorimetry (DSC) Analysis

Table 5 lists the DSC analysis of milk fat and ice melting phase transitions. The results of temperatures and enthalpies for the milk fat phase transition were calculated from the first and second melting curves of all the analyzed cheeses. The first heating curve was obtained during the heating of cheese samples from 5 °C to 70 °C, and the second scan during heating from −40 °C to 70 °C of samples previously cooled to −40 °C. The presence of endothermic peaks proved that part of the milk fat was present at room temperature in the form of crystals in fat globules. The DSC parameters of temperatures and enthalpies calculated from first heating curves (Table 5) showed significant differences between the mean values of temperatures TI_onset_ and TI_peak_ as well as melting enthalpy ΔH_m1_, however, temperature TI_end_ differences were not significant (*p* > 0.05). The highest values of melting enthalpy were measured for cheese from cow’s and frozen sheep’s milk (CSF sample), and the lowest for the cheese from cow’s milk. A second heating of cheese samples enabled the observation of the melting phase transition without any thermal history, thus, the results of temperature and enthalpies differ from those of the first cycle of heating. However, the shape of the second heating curves did not differ for all types of cheese. Based on the DSC results from all the second heating cycles (Table 5) of all types of cheeses, we found that, similarly to the first heating curves, there were differences in all melting temperatures and enthalpies (*p <* 0.05). Only for samples S and SF, were the differences not significant in the case of enthalpy. Generally the results of melting enthalpy followed the pattern from the first heating cycle.

During the second heating, the phase transition of ice melting in the cheese matrix was measured (Table 5). In the case of this phase transition it was possible to measure the amount of water frozen during cooling, which enabled the determination of unfrozen, bound water percentage. Significant differences were observed for temperatures and enthalpy of ice melting. The highest value of enthalpy ΔH_ice_ and higher of temperatures Ti_onset_ and Ti_peak_ for samples of cheese from cow’s milk were obtained. These observations were in agreement with the results of moisture content shown in Table 2, where the highest value of moisture (602.7 g/kg) was noted for cheese made from cow’s milk. Table 5 shows the percentages of unfrozen water in the water fraction (UFW) of cheese, which were calculated according to Equation (2). The lowest percentage of UFW was found in cheese made from cow’s milk, which was due to its lowest protein content (Table 2). In the case of the remaining cheese samples, the UFW level was similar except for cheese made from frozen sheep milk, where the value was lower than for cheese from unfrozen sheep’s milk. The results were also in agreement with the increased water activity (Table 2) from 0.961 for cheese sample S to 0.9773 for cheese sample SF. This indicated the influence of freezing on the binding of water, caused by changes in the protein’s conformations.

Low-field nuclear magnetic resonance (LF NMR) methods can be used to observe the molecular properties of water in cheeses [55]. The values of spin−lattice T1 relaxation times reflected the relationship between bulk water and bound water fractions. As the amount of bulk fraction increased, the value of this relaxation time increased (Table 6). For cow’s milk cheese, the most bulk water was observed compared to the bound fraction. Both fractions were also characterized by much greater molecular dynamics, which manifested in higher values of both components of spin−spin relaxation times. This was directly related to the protein content in milk [56]. Proteins are a natural water-binding biopolymer. Hence, a significantly lower value of this parameter should be obtained for sheep’s milk cheese. The observed shortening of the spin−lattice relaxation times in cheeses containing sheep’s milk after freezing was associated with a significant loss of water, as evidenced by increased a_w_ value. The bound water fraction in cheeses containing sheep’s milk had similar molecular dynamics. This confirmed the role of protein as a substance with water-binding properties. Analysis of the spin−spin relaxation time values describing the molecular dynamics of the bulk fraction (T22), revealed that the addition of previously frozen milk significantly changes the molecular dynamics of this fraction. The increased mobility of the bulk fraction could lead to easier evacuation of water from the system, which in turn, significantly affected the texture parameters.

### 3.5. Investigation of Consumer Perception

Consumers showed the smallest differences between cow’s milk cheese and cheese from the mixture with unfrozen sheep’s milk (Table 7). In general, sheep’s milk pasta filata cheeses were less accepted by consumers than cow’s milk cheeses. Dissatisfaction (dislike) was even greater with frozen milk cheeses. The dislike responses for cheeses made only from sheep’s milk increased from 7% (nonfrozen milk) to 32% (frozen milk).

The main reason for consumer dissatisfaction with cheeses made entirely or only with the addition of frozen milk was not enough of the qualities: refreshing (flavor), elasticity (texture), and shininess (appearance, Table 8). Freezing the milk changed the acidity (aroma) from not enough to too much. In contrast, in the case of sweet milk (aroma), it transitioned from too much to not enough.

The consumer penalty analysis of just-about-right (JAR) did not reveal differences only between cow’s milk cheese and cheese with the addition of unfrozen sheep’s milk. Unfrozen sheep’s milk cheeses generally had not enough: acidity (aroma), refreshing (flavor); and too much: sweet milk (flavor) shininess (appearance) compared to cow’s milk cheeses. Importantly, more than 10% of consumers chose the JAR category.

Faccia et al. [32] found pasta filata cheese made of sheep’s milk compared to cow’s milk had more milk odor and flavor, was less elastic and rubbery (texture during chewing), and was more slippery. This was due to the different behavior of the curds during stretching. They showed porcelainity (external appearance) as a peculiar characteristic, while cow’s cheeses were not shiny. In our study, pasta filata cheeses made from sheep’s milk had (according to the consumer penalty analysis of the JAR) too much shininess (12.37%). Pasta filata Caciocavallo cheese produced by Niro et al. [21] from the mixture of cow’s and sheep’s milk (82:18, *v*/*v*) compared to cow’s cheese was less sweet, saltier, less elastic, more friable and harder. Increased hardness was also demonstrated by Aminifar et al. [57].

## 4. Conclusions

The production of pasta filata cheese from frozen sheep’s milk was not a good alternative to using the raw material. The new cheeses were not fully accepted by consumers. This creates big constraints for development and innovation in the small ruminant dairy sector. Frozen/thawed sheep’s milk did not appear to contribute to the development of innovative and reformulated cheeses. The rationale was the changes in the texture and meltability profile of cheeses made entirely of frozen sheep’s milk or only with 30% in a mixture with cow’s milk. The addition of frozen milk reduced stretching, which was the most characteristic feature of pasta filata cheeses. The freezing of milk makes cheeses, made entirely or with partial addition, harder and brittle, less stretchy and less elastic. These cheeses had the highest values of melting enthalpy measured. The level of unfrozen water in the water fraction was similar except for the cheese made from frozen sheep’s milk, where the value was lower than for cheese from unfrozen sheep’s milk. This result was also justified by increased water activity. Therefore, freezing influenced the binding of water, caused by the changes in protein conformations. At the same time, their flowability increased, which probably had a decisive influence on the overall appearance of the cheeses. The use of frozen milk significantly increased the number of dissatisfied consumers. The consumer penalty analysis of the just-about-right scale showed that freezing milk caused the loss of the refreshing, elasticity and shininess qualities of the produced pasta filata cheeses. Additionally, in cheeses made only from frozen milk, aroma acidity was intensified and the sweet-milk flavor disappeared. Our results can be the basis for research into the use of sheep’s milk as an addition in dairy technologies other than cheese making and for research on the use of frozen sheep’s milk. The results of our research could be an interesting and real opportunity for the dairy industry, allowing for market expansion of sheep’s milk.

## Figures and Tables

**Table 1:** Composition and technological parameters of raw cow’s and sheep’s milk.

Parameters	Cow’s Milk		Sheep’s Milk		SEM
	Mean	P_5_–P_95_	Mean	P_5_–P_95_	
Solids-not-fat (g/kg)	83.4 ^a^	82.9–83.8	121.1 ^b^	120.8–121.3	0.074
Fat (g/kg)	46.3 ^a^	46.1–46.5	86.1 ^b^	85.8–86.4	0.037
Casein (g/kg)	27.1 ^a^	26.8–27.4	41.8 ^b^	41.5–42.0	0.044
Whey protein (g/kg)	6.4 ^a^	6.1–6.7	9.5 ^b^	9.2–9.8	0.048
Lactose (g/kg)	43.6 ^a^	43.3–43.8	47.1 ^b^	46.9–47.4	0.035
Fat/protein	1.4		1.7		
Ash (g/kg)	6.8 ^a^	6.6–7.0	9.1 ^b^	8.9–9.3	0.024
pH	6.64 ^a^	6.60–6.67	6.65 ^a^	6.62–6.67	0.000
Titratable acidity (% lactic acid)	0.162 ^a^	0.160–0.165	0.216 ^b^	0.214–0.218	0.000
Conductivity (mS/cm)	4.12 ^b^	4.10–4.14	3.85 ^a^	3.82–3.89	0.001
Freezing point (°C)	−0.528 ^b^	−0.533–−0.523	−0.571 ^a^	−0.574–−0.568	0.000
Viscosity (mPa∙s)	4.22 ^a^	4.18–4.27	6.71 ^b^	6.68–6.75	0.001
Density, in 20 °C (kg/m^3^)	1.031 ^a^	1.029–1.034	1.037 ^b^	1.033–1.040	0.000
Water activity	0.9838 ^b^	0.9836–0.9839	0.9761 ^a^	0.9759–0.9763	0.000

^a–b^ Means within a row with different superscripts differ (*p* < 0.05); P_5_–P_95_: confidence interval of the mean; SEM: standard error of the mean (*n* = 5).

**Table 2:** Composition and physicochemical properties of pasta filata cheeses from frozen cow’s and sheep’s milk.

Parameters	Pasta Filata Cheese					SEM
	C	CS	CSF	S	SF	
Moisture (g/kg)	602.7 ^c^	568.5 ^b^	569.2 ^b^	488.8 ^a^	491.1 ^a^	0.169
Fat (g/kg)	190.5 ^a^	188.6 ^a^	189.7 ^a^	189.3 ^a^	191.4 ^a^	0.078
Fat/dry matter (*w*/*w*)	0.48	0.44	0.44	0.37	0.38	
Protein (g/kg)	174.2 ^a^	204.1 ^b^	198.7 ^b^	266.8 ^c^	270.3 ^c^	0.069
Protein/dry matter (*w*/*w*)	0.44	0.47	0.46	0.52	0.53	
Protein/fat (*w*/*w*)	0.9	1.1	1.1	1.4	1.4	
Salt (g/kg)	0.47 ^a^	0.46 ^a^	0.45 ^a^	0.46 ^a^	0.46 ^a^	0.000
pH	5.13 ^a^	5.12 ^a^	5.11 ^a^	5.12 ^a^	5.12 ^a^	0.000
Acidity (% lactic acid)	0.702 ^a^	0.709 ^a^	0.695 ^a^	0.716 ^a^	0.702 ^a^	0.000
Water activity	0.9605 ^a^	0.9586 ^a^	0.9714 ^b^	0.9614 ^a^	0.9773 ^b^	0.000

^a–c^ Means within a row with different superscripts differ (*p* < 0.05); SEM: standard error of the mean (*n* = 5); C: from cow’s milk; CS: from cow’s and unfrozen sheep’s milk in proportion 70:30; CSF: from cow’s and frozen sheep’s milk in proportion 70:30; S: from unfrozen sheep’s milk; SF: from frozen sheep’s milk.

**Table 3:** Texture parameters of pasta filata cheeses from cow’s and sheep’s frozen milk.

Parameters	Pasta Filata Cheese					SEM
	C	CS	CSF	S	SF	
Extensibility force (g)	35.4 ^c^	33.8 ^b^	37.4 ^d^	26.6 ^a^	42.5 ^e^	0.069
Stretching (mm)	127.8 ^a^	131.4 ^b^	129.8 ^a^	132.3 ^b^	133.1 ^b^	0.082
Hardness (g)	264.3 ^b^	259.7 ^b^	322.8 ^c^	188.7 ^a^	364.8 ^d^	0.042
Brittleness (mm)	108.1 ^a^	113.9 ^b^	108.9 ^a^	122.7 ^c^	111.0 ^b^	0.059
Cutting force (g)	34.9 ^c^	30.6 ^b^	41.4 ^d^	23.7 ^a^	54.8 ^e^	0.045

^a–e^ Means within a row with different superscripts differ (*p* < 0.05); SEM: standard error of the mean (*n* = 5); C: from cow’s milk, CS: from cow’s and unfrozen sheep’s milk in proportion 70:30; CSF: from cow’s and frozen sheep’s milk in proportion 70:30; S: from unfrozen sheep’s milk; SF: from frozen sheep’s milk.

**Table 4:** Meltability (M) and flowability (F) parameters of pasta filata cheeses from cow’s and sheep’s frozen milk.

Parameters	Pasta Filata Cheese					SEM
	C	CS	CSF	S	SF	
M—Tube Test (mm)	7.2 ^b^	8.6 ^c^	6.9 ^b^	11.1 ^d^	6.4 ^a^	0.043
M—Schreiber Test (scale 0–10)	3.3 ^a^	3.8 ^b^	3.2 ^a^	5.2 ^c^	3.2 ^a^	0.037
F—Oiling–off (%)	3.13 ^a^	2.92 ^a^	11.56 ^c^	4.21 ^b^	51.85 ^d^	0.001

^a–d^ Means within a row with different superscripts differ (*p* *<* 0.05); SEM: standard error of the mean (*n* = 5); C: from cow’s milk; CS: from cow’s and unfrozen sheep’s milk in proportion 70:30; CSF: from cow’s and frozen sheep’s milk in proportion 70:30; S: from unfrozen sheep’s milk; SF: from frozen sheep’s milk.

**Table 5:** Differential scanning calorimetry (DSC) parameters of pasta filata cheeses from cow’s and sheep’s unfrozen and frozen milk.

Parameters	Pasta Filata Cheese				
	C	CS	CSF	S	SF
First heating Temperature					
TI _on_ (°C)	11.78 ^b^	11.15 ^ab^	10.27 ^ab^	11.78 ^b^	8.625 ^a^
TI_peak_ (°C)	15.97 ^c^	13.15 ^a^	16.88 ^d^	13.85 ^ab^	14.69 ^b^
TI_end_	37.71 ^a^	37.57 ^a^	38.97 ^a^	36.54 ^a^	37.36 ^a^
Enthalpy ΔH_m1_ (J/g of fat)	36.06 ^a^	44.18 ^ab^	84.43 ^d^	57.72 ^bc^	67.15 ^cd^
Second heating Temperature					
TII_on_ (°C)	12.18 ^c^	7.92 ^a^	12.61 ^c^	7.43 ^a^	10.60 ^b^
TII_peak_ (°C)	15.88 ^c^	13.12 ^a^	16.42 ^c^	13.29 ^a^	14.98 ^b^
T_end_	36.24 ^b^	34.01 ^a^	36.59 ^b^	34.17 ^a^	35.99 ^b^
Enthalpy ΔH_m2_ (J/g of fat)	41.95 ^a^	70.88 ^b^	105.82 ^d^	85.39 ^c^	80.15 ^c^
Ice melting Temperature					
Ti_onset_ (°C)	−4.14 ^d^	−13.80 ^c^	−13.27 ^c^	−19.92 ^a^	−16.50 ^b^
Ti_peak_ (°C)	−0.11 ^c^	−7.30 ^b^	−6.54 ^b^	−13.24 ^a^	−7.57 ^b^
Enthalpy ΔH_ice_ (J/g of water)	232.74 ^c^	100.36 ^a^	99.77 ^a^	90.16 ^a^	153.85 ^b^
Unfreezable water (g/100 g of water)	29.97 ^a^	69.80 ^c^	69.98 ^c^	72.87 ^c^	53.7 ^b^

^a–d^ Means within a row with different superscripts differ (*p* < 0.05); C: from cows milk; CS: from cows and unfrozen sheep milk in proportion 70:30; CSF: from cows and frozen sheep milk in proportion 70:30; S: from unfrozen sheep milk; SF: from frozen sheep milk.

**Table 6:** The values of the spin−lattice T_1_ and both components of the spin−spin T_2_ relaxation times in pasta filata cheeses from cow’s and sheep’s frozen milk.

Relaxation Parameters	Pasta Filata Cheese					SEM
	C	CS	CSF	S	SF	
T_1_ (ms)	279.3 ^d^	228.4 ^c^	120.9 ^a^	125.9 ^b^	121.3 ^a^	0.206
T_21_ (ms)	24.1 ^c^	10.8 ^a^	10.7 ^a^	10.9 ^ab^	11.3 ^b^	0.050
T_22_ (ms)	164.1 ^e^	32.2 ^a^	59.4 ^b^	61.7 ^c^	64.1 ^d^	0.032

^a–e^ Means within a row with different superscripts differ (*p* < 0.05); SEM: standard error of the mean (*n* = 5); C: from cow’s milk; CS: from cow’s and unfrozen sheep’s milk in proportion 70:30; CSF: from cow’s and frozen sheep’s milk in proportion 70:30; S: from unfrozen sheep’s milk; SF: from frozen sheep’s milk.

**Table 7:** Sensory acceptability of pasta filata cheeses from cow’s and sheep’s frozen milk.

		Pasta Filata Cheese				
		C	CS	CSF	S	SF
9	Like extremely (%)	2.06	5.15	0	0	0
8	Like very much (%)	27.84	40.21	1.03	2.06	0
7	Like moderately (%)	30.93	35.05	28.87	40.21	0
6	Like slightly (%)	11.34	1.03	39.18	21.65	17.53
5	Neither like nor dislike (%)	27.84	16.49	22.68	25.77	50.52
4	Dislike slightly (%)	0	2.06	6.19	3.09	15.46
3	Dislike moderately (%)	0	0	2.06	7.22	12.37
2	Dislike very much (%)	0	0	0	0	4.12
1	Dislike extremely (%)	0	0	0	0	0
Skewness		0.66	1.28	1.09	1.22	2.02
*p*–value		0.005	0.004	0.009	0.018	0.003
SD		13.41	15.48	14.61	14.16	15.93
CV		124.38	143.65	135.55	131.36	147.78
Dislike responses (%)		0	2.06	8.25	10.31	31.95

SD: standard deviation; CV: coefficient of variation; C: from cow’s milk; CS: from cow’s and unfrozen sheep’s milk in proportion 70:30; CSF: from cow’s and frozen sheep’s milk in proportion 70:30; S: from unfrozen sheep’s milk; SF: from frozen sheep’s milk.

**Table 8:** Consumer penalty analysis of the just-about-right (JAR) diagnostic attributes of pasta filata cheeses from cow’s and sheep’s frozen milk.

			Pasta Filata Cheese				
			C	CS	CSF	S	SF
Aroma	acidity	not enough	–	–	–	14.43	–
		too much	–	–	–	–	13.40
Flavor	refreshing	not enough	–	–	16.49	19.59	35.05
		too much	–	–	–	–	–
	sweet milk	not enough	–	–	–	–	14.43
		too much	–	–	–	11.34	–
Texture	elasticity	not enough	–	–	17.53	–	47.42
		too much	–	–	–	–	–
	smoothness	not enough	–	–	–	–	–
		too much	–	–	–	–	–
Appearance	shininess	not enough	–	–	17.53	–	24.74
		too much	–	–	–	12.37	–

(–): indicates that less than 10% of consumers chose that JAR category; C: from cow’s milk; CS: from cow’s and unfrozen sheep’s milk in proportion 70:30; CSF: from cow’s and frozen sheep’s milk in proportion 70:30; S: from unfrozen sheep’s milk; SF: from frozen sheep’s milk.

## Data Availability

The authors confirm that the data supporting the findings of this study are available within the article.
